# Retrospective genomics highlights changes in genetic composition of tiger sharks (*Galeocerdo cuvier*) and potential loss of a south-eastern Australia population

**DOI:** 10.1038/s41598-022-10529-w

**Published:** 2022-04-21

**Authors:** Alice Manuzzi, Belen Jiménez-Mena, Romina Henriques, Bonnie J. Holmes, Julian Pepperell, Janette Edson, Mike B. Bennett, Charlie Huveneers, Jennifer R. Ovenden, Einar E. Nielsen

**Affiliations:** 1 National Institute of Aquatic Resources, Technical University of Denmark, Vejlsøvej 39, 8600 Silkeborg, Denmark; 2 School of Science, Technology & Engineering, University of the Sunshine Coast, Sippy Downs, QLD 4556 Australia; 3 Pepperell Research and Consulting, PO Box 1475, Noosaville DC, QLD 4566 Australia; 4 Queensland Brain Institute, The University of Queensland, Brisbane, QLD 4072 Australia; 5 School of Biomedical Sciences, The University of Queensland, Brisbane, QLD 4072 Australia; 6 College of Science and Engineering, Flinders University, Adelaide, SA 5001 Australia; 7 Molecular Fisheries Laboratory, School of Biomedical Sciences, The University of Queensland, Brisbane, QLD 4072 Australia

**Keywords:** Genetics, Population genetics, Marine biology

## Abstract

Over the last century, many shark populations have declined, primarily due to overexploitation in commercial, artisanal and recreational fisheries. In addition, in some locations the use of shark control programs also has had an impact on shark numbers. Still, there is a general perception that populations of large ocean predators cover wide areas and therefore their diversity is less susceptible to local anthropogenic disturbance. Here we report on temporal genomic analyses of tiger shark (*Galeocerdo cuvier*) DNA samples that were collected from eastern Australia over the past century. Using Single Nucleotide Polymorphism (SNP) loci, we documented a significant change in genetic composition of tiger sharks born between ~1939 and 2015. The change was most likely due to a shift over time in the relative contribution of two well-differentiated, but hitherto cryptic populations. Our data strongly indicate a dramatic shift in the relative contribution of these two populations to the overall tiger shark abundance on the east coast of Australia, possibly associated with differences in direct or indirect exploitation rates.

## Introduction

Intraspecific genetic diversity is essential for long-term population persistence to avoid the negative effects of inbreeding^1^, to buffer against environmental variation in time and space^2^, and to ensure adaptability to a changing environment^3^. High genetic diversity may also have positive ecosystem effects by promoting productivity, abundance, and stability of community structure^4–6^. Thus, species with many discrete populations with different genetic adaptations, including life history strategies, are assumed better at withstanding exploitation and environmental variation, than a single panmictic population. Intraspecific diversity protection is a specified objective of the Convention on Biological Diversity (CBD; www.cbd.int), but is rarely an integral part of monitoring activities, in particular for species that are not critically endangered^7,8^. Indeed, the lack of dedicated sampling programs and the many species requiring monitoring makes it logistically impossible to oversee intra-specific diversity in most instances. In addition, the difficulties associated with the use of high-resolution genetic methods when sample sizes are low or when tissue samples vary in age, composition and provenance^9^ also hinder our ability to assess intra-specific diversity.

The use of DNA extracted from museum specimens, in combination with modern molecular analytical tools, has revolutionized the ability to assess genetic changes at contemporary time scales^10,11^. Studies on processes such as intra-population loss of diversity, adaptive change caused by evolutionary drivers in the environment, as well as the movement, decline, or extirpation of populations through time and space can now be undertaken^5,12^. In the marine realm, there are numerous examples of local population reductions and extinctions, in particular for large sharks and rays^13,14^, but closely coupling these incidences with genetic and genomic data is challenging due to the lack of temporal genetic data for elasmobranch species. Spatiotemporal genetic samples (i.e. obtained across several locations and years^15^) can be used to test for changes in distribution of elasmobranch populations including potential extirpations and replacements, as well as intra-population changes in levels of genetic variability, and putative adaptive genetic changes in the timeframe of recent environmental changes or exploitation. This is of significant interest with respect to populations of large sharks, where there is a strong need for identification of genetic populations and their historical trajectories, as they represent both the relevant unit for evolution and management^9,16^ in order to contribute significantly to short-term sustainable exploitation as well as long-term protection of intra-specific biodiversity. Failing to identify and incorporate biologically-discrete populations in management may cause overharvesting of particular (cryptic) populations or population segments, resulting in extinction or reduction of genetic diversity within populations^4,7^.

The tiger shark (*Galeocerdo cuvier*) is one of the world’s largest sharks, with a circumglobal distribution in tropical and sub-tropical waters^17,18^. Throughout its range, the species is exploited by multiple users, from target and bycatch in commercial, artisanal, and recreational fisheries^19,20^, to shark control operations to improve bather safety^21,22^, which have been linked to on-going population decline and reduced average size of individuals^22–24^. The species is globally listed as Near Threatened on the International Union for the Conservation of Nature’s (IUCN) Red List of Threatened Species due to a suspected decline of ~ 30% over the past ~ 60 years^25^. This general decline covers variable regional population trends ranging from relatively stable abundances, e.g. North-western Atlantic^26^, to severe local depletions as observed in the Red Sea, Eritrea, Iranian Gulf, as well India and Pakistan^27^. In the Arabian Seas on-going local reported declines of 30–50% over three generations led the species to be locally assessed as Vulnerable^27^. Of greater concern, however, is the reported 74% decline in catch per unit effort (CPUE) for tiger sharks off Queensland (eastern Australia) over the past 25 years, which was accompanied by a 21% decline in the average size of individuals^24^. Thus, despite its cosmopolitan nature and dispersal potential, local depletions appear to be relatively common in the species, suggesting some degree of population isolation could occur. However, there is still a lack of adequate spatially resolved data to understand regional population trends of tiger sharks as they are commonly caught in unreported fisheries. Current assessments of the tiger shark population structure have been based on microsatellite and mitochondrial genetic markers, and have demonstrated a clear inter-basin genetic split between the Atlantic and Indo-Pacific Oceans, but a general lack of intra-basin population structuring^28–30^. Although this may be a true biological characteristic of the species, it could also reflect analytical challenges related to obtaining sufficient samples with good spatial coverage and the application of low-resolution genetic markers. Thus, in a recent review of research priorities, the development of high-resolution SNPs (Single Nucleotide Polymorphisms) to further resolve tiger shark meta-population structure was strongly recommended^31^. Moreover, temporal genetic information to assess the anthropogenic effects of exploitation is virtually absent. Such temporal genetic data are necessary to determine both intra-population changes in diversity by genetic drift, migration and selection, but also extent of population displacement or extirpation. Due to its Near Threatened status, potential for over-exploitation and risk to human lives, tiger shark management and conservation actions are high on the public agenda^32^. As large sharks are highly mobile, management has been primarily focused on international or regional policies, with limited value of enforcing strong local “domestic” regulations^16^. However, the detection of fine-scale population structure and the effects of local depletion or extirpation on intraspecific genetic diversity could lead to a general paradigm shift in large shark management from a global or regional perspective to a much stronger local focus.

Here, we investigated a possible link between the reported decline in tiger shark numbers and local genetic diversity off the east coast of Australia. While mating system can affect effective population size^33,34^ and declines in genetic diversity can be moderated by gene flow^35^, genetic diversity has been shown to be linked to effective population size. Indeed, a reduction in population size (e.g. bottleneck) can lead to a rapid decline in heterozygosity and allele diversity^36^. Such relationship has also been reported in empirical studies (e.g.^37,38^). We chose east coast Australia because of documented reductions in tiger shark CPUE in that region and because samples from this region are relatively abundant across both spatial and temporal scales; i.e. tiger sharks samples were available along the eastern Australian coastline and from as early as ~1939 to 2015. This provided an unparalleled opportunity to assess how the alleged population decline may have affected the genetic composition of the species over the past century. In order to collect genetic data over the widest spatial and temporal range, we extracted DNA from tissue samples taken from shark jaws archived in museums and private collections, mostly retained as trophies from fishing competitions, and performed retrospective genomic analyses^39^. As these trophies are listed with local game-fishing clubs, their size, catch date and location are well documented.

Tiger sharks in Australia are thought to be part of a large Indo-Pacific population^28^, and satellite-tag tracking studies have revealed extensive movements up to several thousand kilometres in the region^40,41^ with some individuals seen moving as far as New Caledonia (maximum reported distances: of 1141 and 1800 km)^42,43^. In this context, we hypothesised that tiger sharks in eastern Australia, form a single panmictic population, from tropical Queensland to temperate Victoria^42,44^. Therefore, by employing a local spatiotemporal approach, our study aimed to assess if (i) tiger sharks in eastern Australia are indeed panmictic, (ii) reported declines are related to possible population structure in the species and if so, (iii) whether population distributions and relative abundances have remained stable over the last 100 years. This is the first study of its kind, not only for the region and tiger sharks, but also for elasmobranchs in general.

## Results

### Bioinformatics pipeline and data filtering

**Figure 1:**
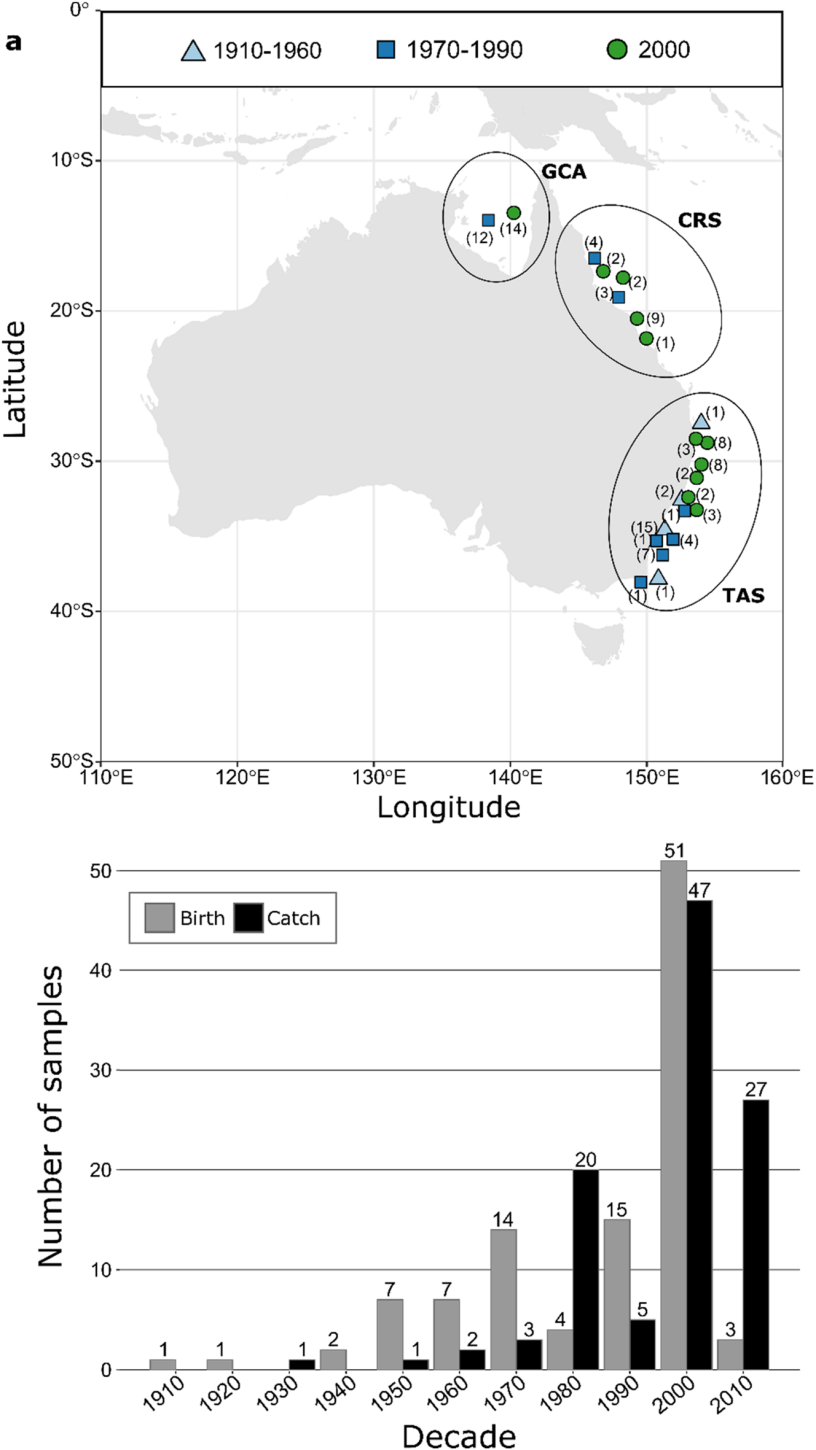
Sampling locations and distribution through time and space. (**a**) Samples distribution along the east coast of Australia. Samples are grouped by decades of birth (1910–1960, 1970–1990 and 2000) as explained in the text and the colours identify the three time-periods. Group names refer to the three major regions where samples were collected: Gulf of Carpentaria (GCA), Coral Sea (CRS) and Tasman Sea (TAS). (**b**) The histogram shows the difference between decade of catch and calculated decade of birth and associated sample numbers, as reported above each bar. Grey bars identify the calculated years of birth, while black numbers refer to the years of catch.

Sequencing yielded an average of 2,833,138 reads per individual, with slightly lower numbers for historical jaw samples (median 1,973,493) than contemporary tissue samples (median 2,301,001). After running the bioinformatics pipeline, 78% of the original sequences went into transcriptome mapping. All samples showed a very low percentage of contaminants, confirming the validity of the capture strategy for sequencing enrichment with shark DNA. An average of 0.12% of the cleaned reads was of mitochondrial origin and excluded from further analysis, except for the few cases when it was used for species confirmation. Out of the 20,000 catshark derived baits, 4544 (22.72%) were post-hoc mapped back to the tiger shark transcriptome^45^ covering 4137 scaffolds. Of these, 4143 had captured reads with a depth of coverage higher than non-target regions. Scaffolds with a bait had an average coverage of 68.5×, while scaffolds from off-target regions had an overall average depth of 40.7× (35.8× for historical and 43.4× for contemporary samples). Coverage was higher and less variable in contemporary than in historical samples. Single nucleotide polymorphisms (SNPs) were called from all transcriptomic sequences and we identified 35,061 raw SNP variants for 122 samples in 2978 reference scaffolds. After filtering, 4580 SNPs remained. SNPs with significant departures from Hardy–Weinberg Equilibrium (HWE) were removed to produce a final dataset consisting of 1840 SNP loci genotyped in 115 samples from *G. cuvier* specimens caught between ~1939 and 2015. In addition, we removed samples that had higher levels of missing data (below 70% call rate), had lack of length/weight information that did not allow to calculate the age, or had erroneous species labelling (identified as a different species), that could indicate a possible contamination with reads from other shark species. The final dataset thus contained 106 samples (Fig. 1, Table S1). Very low levels of DNA damage was observed, confirming DNA was well preserved in jaws^39^, at least for the relatively short time period (~ 80 years) compared to true ancient DNA (aDNA) studies. Thus, it is highly unlikely that the final SNPs represent artefacts due to deamination or other DNA damage in the historical jaw samples.

### Data analysis for temporal and spatial genomic variability

**Figure 2:**
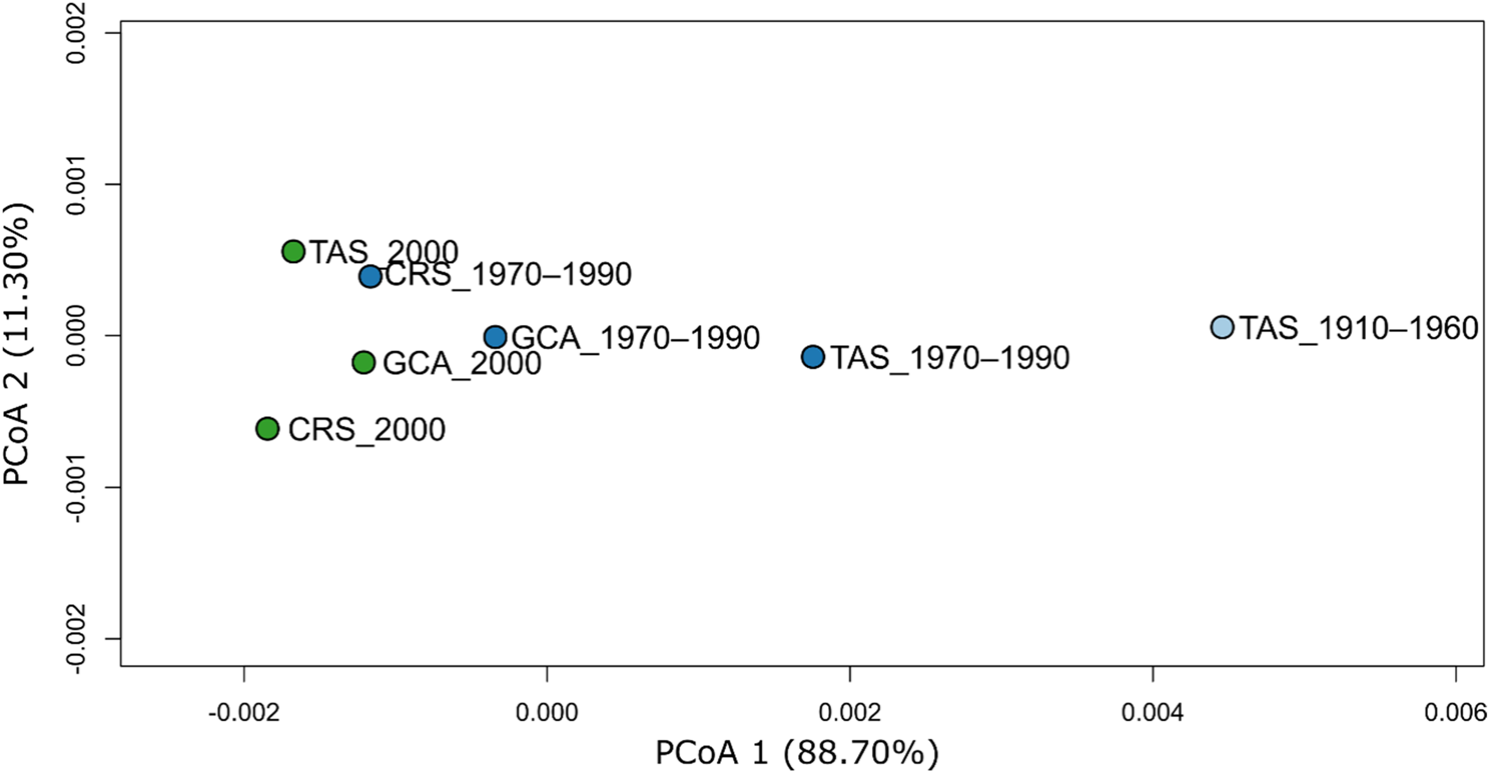
Principal Coordinates Analysis (PCoA) of mean pairwise FST’s between spatiotemporal tiger shark samples from eastern Australia. Groups are based on back-calculated ages and refer to: Gulf of Carpentaria (GCA), Coral Sea (CRS) and Tasman Sea (TAS). The axes report the percentage of variance explained.

**Figure 3:**
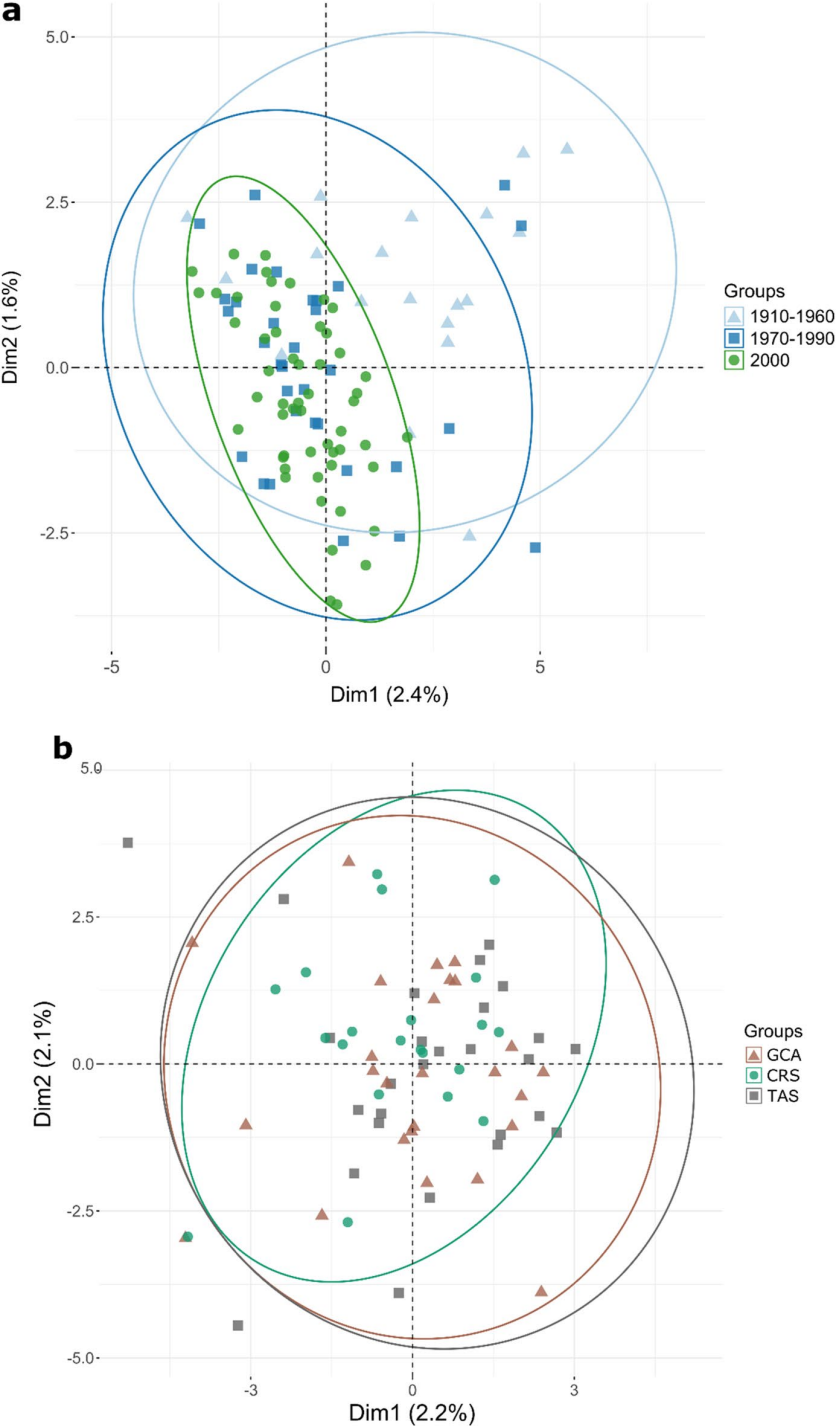
Principal Component Analysis (PCA) by time periods and locations. (**a**) PCA of all individual genotypes for the samples with back-calculated age of birth and (**b**) PCA of contemporary samples based on decade of catch (2000–2010) covering the Gulf of Carpentaria (GCA), Coral Sea (CRS) and Tasman Sea (TAS).

**Figure 4:**
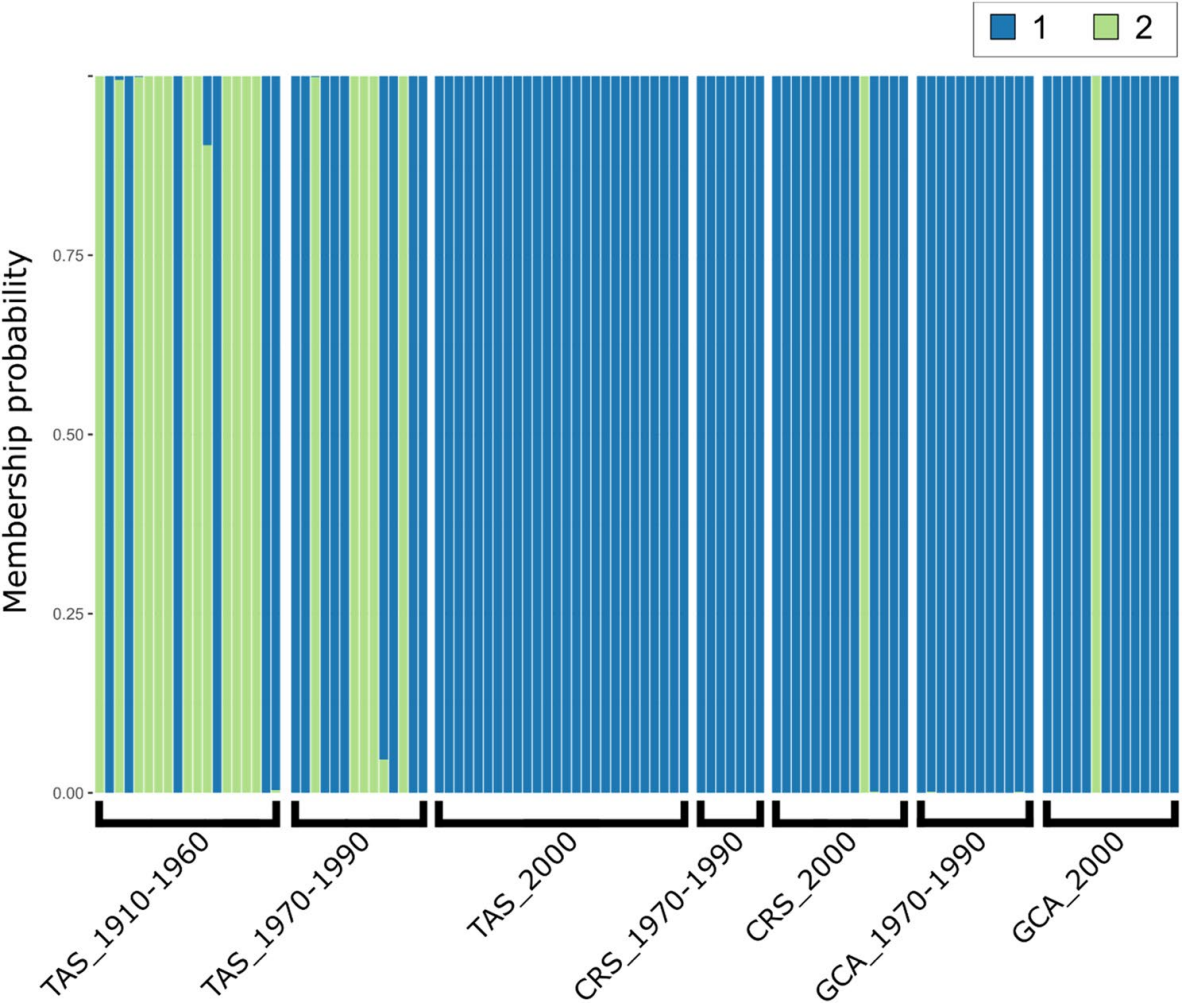
Discriminant Analysis of Principal Components (DAPC) for K = 2. The plot illustrates the spatiotemporal occurrence of individuals from the two hypothesized clusters in time and space. Samples grouped by time and space are labelled along the x-axis, only collections encompassing more than six samples were included. The y-axis reports the membership probability of each sample to belong of either clusters (‘1’ in blue and ‘2’ in green).

**Figure 5:**
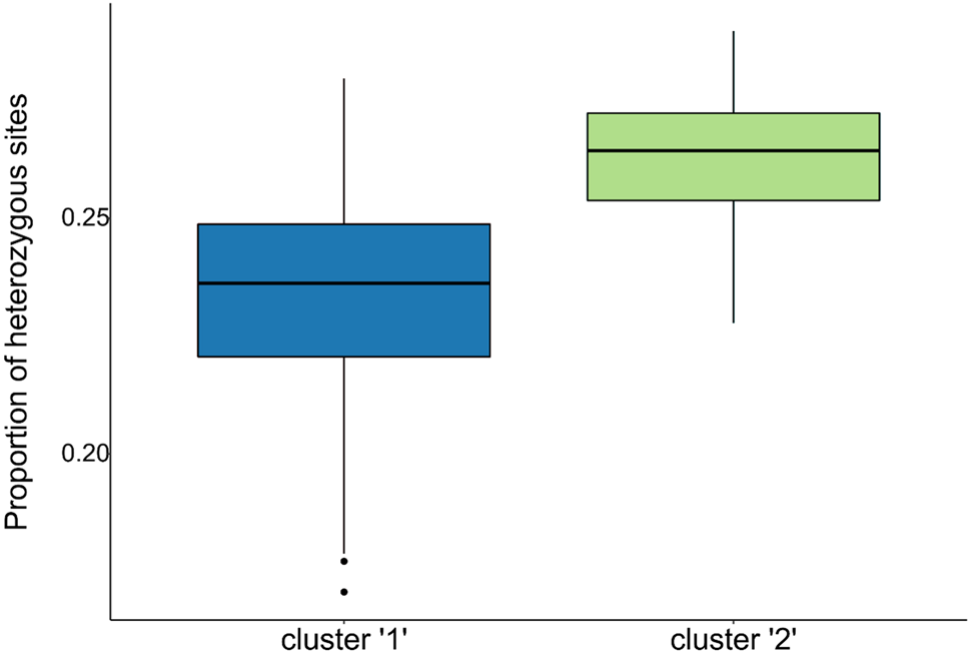
Boxplot of the average proportion of heterozygous SNPs loci for the two clusters. Average proportion of heterozygous SNP loci over total loci genotyped for the two identified clusters. Cluster 1 is composed of mainly contemporary and northern samples, while cluster 2 individuals are almost exclusively found in southern historical samples (see Fig. 4 for explanation).

To examine the stability of patterns over time, we used a temporal genetic analysis based on the back-calculated decade of birth of tiger shark individuals, which showed clear evidence of temporal genetic differentiation. This was apparent for the temporal dataset as a whole (Table 1), where pairwise F_ST_ estimates increased with time. Temporal differentiation was also apparent for the Tasman Sea samples alone, where the majority of the oldest historical samples originated (Table S2). In contrast, contemporary tiger shark samples (2000 and 2010) from the Gulf of Carpentaria (GCA), Coral Sea (CRS) and Tasman Sea (TAS), showed little evidence of genetic structuring, with estimated non-significant pairwise F_ST_ values of 0.006 between GCA and CRS, and 0.001 between CRS and TAS (Table S3). A Principal Coordinates Analysis (PCoA) of spatiotemporal F_ST_ values (Fig. 2) showed a separation of samples along axis 1, explaining 88.7% of the variation. The 1910–1960 TAS samples were the most distinct, with the 1970–1990 TAS samples intermediate between the TAS oldest samples and a cluster of the remaining samples. The Principal Component Analysis (PCA) of all spatiotemporally collected individuals (Fig. 3a) supported the genetic differentiation of historical samples as they clustered differently, with a proportion of the individuals forming a relatively distinct cluster from the contemporary samples. In contrast to Fig. 3a, an individual based PCA of spatially-collected contemporary samples only (Fig. 3b) did not display any apparent clustering of individuals according to location. The presence of a distinct group of individuals in the historical samples was supported by the results from the Discriminant Analysis of Principal Components (DAPC), where the Bayesian Information Criterion (BIC) analysis and the k-means algorithm identified K = 2 as the number of clusters that best explains the structure in the dataset (Fig. 4). Further analytical support for two populations was given by the results of the *snmf* algorithm (LEA package) that also identified K = 2 as the most likely number of clusters (Fig. S1). The DAPC analysis showed ~ 70% (13 of 19) of ‘cluster 2’ individuals in the 1910–1960 TAS samples, ~ 36% (5 of 14) in the 1970–1990 samples and a lack among the TAS samples from 2000. For the GCA samples, only a single ‘cluster 2’ individual (1 of 14) was found (2000). Likewise, only one ‘cluster 2’ individual (1 of 14) was found among the CRS 2000 samples. The Fisher exact test confirms that the change over time was statistically significant with all the comparisons between historical versus contemporary collections (TAS_1910–1960 vs TAS 1970–1990, TAS 1910–1960 vs TAS_2000, and TAS_1970–1990 vs TAS_2000) being significant (p-val = 0.041, 0.00001 and 0.003 respectively). Patterns of Site Frequency Spectra (SFS) and the pi and Watterson theta indices (Fig. S2, Table S4) showed that older collections and contemporary collections behaved similarly with no evident bias (e.g. excess of singletons) in older samples. When pooling all putative ‘cluster 1’ and ‘cluster 2’ individuals across samples, the resulting mean F_ST_ between the two population groups was 0.013, thus substantially higher than between any pair of spatiotemporal samples, further supporting a mixed populations hypothesis. The distribution of F_ST_ values across loci showed that a high number of loci (Fig. S3) contributed to the differentiation, suggesting that genetic differences between the two groups were not caused by technical artefacts or contemporary evolution at one or a few loci. However, while BayeScan did not detect any outliers, pcadapt identified 14 possible outlier loci between the two clusters. Most of these loci (9 of 14) showed an overall higher allele frequency for ‘cluster 2’. The mean level of heterozygosity in the two groups was statistically different (Fig. 5), with 0.23 (± 0.01) for ‘cluster 1’ and 0.26 (± 0.01) for ‘cluster 2’. Average missing data was lower for ‘cluster 1’ than ‘cluster 2’ (0.35% and 1.14%, respectively), likely reflecting the average age of samples. However, there was no correlation between mean individual heterozygosity and proportion of missing data using a linear model (*p* = 0.25; R^2^ = 0.003).

**Table 1:** Pairwise F_ST_ values between temporal collections.

	1910–1960	1970–1990	2000
1910–1960	–	0.000*	0.000*
1970–1990	0.004*	–	0.062
2000	0.007*	0.001	–

Pairwise F_ST_ values (lower diagonal) and p-values (upper diagonal) to estimate overall genetic differentiation between temporal samples of tiger sharks from east coast Australia. Sample sizes per time period are: 1910–1960 (N = 19), 1970–1990 (N = 33), 2000 (N = 54).

The star symbol (*) identifies significant values.

## Discussion

This is the first study to demonstrate that archived samples in combination with modern genomic tools can reveal temporal changes in biodiversity in large sharks, which otherwise would have remained unnoticed. We identified genomic differences, consistent with two hitherto unidentified populations, at a relatively local scale (Eastern Australia) for a large migratory shark, the tiger shark. However, this pattern was only evident when historical samples were included, suggesting the displacement or extirpation of a local population. This conclusion is based on the findings that the genetic clustering of our spatiotemporal genotypes provided best statistical support for two population groups, with downstream analysis confirming that these units are genetically distinct and contain different levels of genetic diversity. One of the identified populations was most abundant in the oldest and southern-most samples (i.e. Tasman Sea), and almost completely absent from contemporary (including southern samples) and northern-most samples. We propose that the most parsimonious explanation for this absence is a shift in the relative population abundance likely associated with human fishing activities. Our results mirror the findings of Brown and Roff^46^ that reported a major decline in abundance of tiger sharks over three generations off the Queensland coast, with greater declines detected at the southern sites. However, we recognise that a number of factors could have partially contributed to our observations and uncertainties around this apparent dramatic shift in abundance, which we discuss below.

Our findings are unlikely to be the result of technical issues associated with the use of historical DNA (hDNA). In general, capture sequencing of hDNA has a lower sequencing depth and coverage leading to fewer mapped reads and thus more missing data with increasing sample age^47^, both of which could affect downstream population genomic inferences^48–50^. Although our historical samples presented fewer reads than the contemporary samples, the level of missing data was generally low, even for the oldest samples (maximum level of missing data allowed was 5% per SNP). For example, in ‘cluster 2’ all but two individuals had less than 5% missing data (average of 1.14%, median 0.14%), while all but two individuals in ‘cluster 1’ had less than 1% missing data (average 0.35%, median 0.00%). ‘Cluster 2’ individuals generally showed a higher level of heterozygosity than individuals from ‘cluster 1’, which is the opposite of the expected pattern of reduced individual heterozygosity due to low coverage caused by allelic drop-out^42^. The opposite pattern of increased diversity due to a high occurrence of singletons caused by DNA degradation was also implausible due to the filtering, only including SNPs with a minimum allele count of three in the dataset. In addition, the genetic differentiation that we observed between clusters over time was not caused by a few spurious high-differentiated loci (which could be the result of a technical issue in the genomic pipeline), but was found to be spread across the transcriptome. Moreover, if ‘cluster 2’ genotypes were artefacts created by poor quality DNA, this would still fail to explain the complete absence of those genotypes in the historical northern samples with equal DNA quality (GCA). The observed pattern of differentiation is consistent with a scenario of genetic drift accumulated across the genome, at an evolutionary time scale in two semi-independent populations of tiger sharks, as clearly indicated by the genetic cluster analysis and the transcriptome-wide patterns of differentiation (i.e. differentiation was driven my multiple loci spread throughout the transcriptome). Further strength to this hypothesis come from the results obtained by the two independent clustering methods (DAPC and *snmf* function in LEA), representing different algorithm and population genetic models/assumptions. Both approaches consistently identified the most likely presence of a distinct cluster in the older southern samples, which was not detectable in the most recent/northern collections. Thus, the relatively large differentiation between the two putative population clusters, the differences in their levels of heterozygosity, and the finding that not all historical Tasman Sea samples clustered together, renders the alternative hypothesis of very strong short-term genetic drift in a single panmictic population distributed across the central Indo-Pacific less plausible**.** In summary, the reported temporal genetic differentiation is unlikely to be caused by artefacts in sequencing and in the bioinformatics pipeline, by historical genetic drift in a single panmictic population, or by strong temporal genetic signatures of intra-population environmental selection (which would imply differentiation to be driven by a few loci).

The use of material from historical collections together with genomic-scale analyses provided a relatively large sample size and sufficient statistical power for individual-based cluster analyses, presenting a unique window to explore population composition of tiger sharks in the past. The collection of historical and contemporary samples from the east coast of Australia comprised a huge logistic effort and, at least for the oldest specimens, represents the majority of high quality samples available and practically possible to sample in the community to date. Our samples thus provide relatively solid evidence of changes in population abundance, but still are unlikely to provide the full picture of genetic variation across space and time in tiger sharks off eastern Australia, in particular with respect to population mixing. Although location of catch was accurate, the sampling effort was mainly opportunistic, and thus variation in composition of samples with respect to age and sex, as well as season of sampling and method, may have influenced our results. Unfortunately, there is little information on sex for the historical samples and thus this assumption cannot be directly proven.

Previous tagging studies investigating tiger shark dispersal have shown complex movement patterns including large-scale migrations, seasonal residency, and sex- and size-based dispersal or partial migrations^42,51–58^. In eastern Australia, the movements of tiger shark are closely related to water temperature^42,58^. Prey densities associated with these gradients may also drive adult shark movements to regularly return to specific areas to feed, i.e. established turtle nesting sites^58^. As a species that undergoes ontogenetic diet shift to larger-bodied^45^ prey items as they mature^59^, this could also partly explain a higher occurrence of the local (‘cluster 2’) population in the historical Tasman Sea samples, which were predominately large trophy specimens. However, little is still known about the use of space by tiger sharks, particularly those that are sexually mature, and there is some suggestion that reproductive cues associated with changes in thermal gradient may also drive movements of large females toward the insular regions of the Pacific Ocean^58^.

Sampling the same individual twice or kin sampling might skew the genetic diversity estimated^60^. However, these were not an issue in our study as, firstly, samples were obtained from dead individuals, and secondly, our kinship analysis did not show related individuals in our samples. Despite possible sampling-associated uncertainties, the observed patterns of temporal genetic divergence are clear. Firstly, our results based on contemporary samples are consistent with two recent studies that detected a single genetic population off the Australian east coast^28,29^ and are also compatible with the present understanding of population structure in the Indo-Pacific^29,30^. Secondly, the presence of the two identified cryptic groups was not random in space and time. Individuals from ‘cluster 2’ were almost exclusively detected in the southern part of the species’ distribution, most abundant in the oldest samples, and almost absent from contemporary samples. Consequently, we hypothesize tiger sharks in east Australian waters consisted of at least two populations in the past, but likely comprise a single population now. This may sound counterintuitive in the light of satellite tag-tracking studies that have shown evidence of individuals migrating over 1000 km^23,43,61^. However, large migrations and local populations at fine geographical scales are not mutually exclusive since dispersal events may be related to foraging and not reproductive purpose hence contributing differently to a population genetic make-up. Thus, evidence of fine-scale structure could be linked to basic “triangle migrations^16,62^ of fishes between parturition sites and juvenile and adult habitats^63^. Partial migration, ubiquitous among animal populations, and ontogenetic variation in dispersive behaviour, documented in tiger sharks^53,54,64^), might have also affected estimate of genetic diversity. Indeed, pooling of life stages, i.e. across sizes, during data analysis can obscure estimates of stock structure and dispersal^65^. This is, however, unlikely to be an issue in our study because we were able to identify clear stock structure regardless of the majority of our samples being from large tiger sharks known to be the most dispersive^54,57,64^.

The observed pattern of population structure suggests a southern Australian distribution of one of the populations (‘cluster 2’), with the apparently more abundant population (‘cluster 1’) currently found throughout the entire distribution, with a previous limited intrusion to southern New South Wales (based on the small number of individuals in ‘cluster 1’ from the historical TAS samples). It is possible that the southerly population (‘cluster 2’) was a coastal and more resident ecotype and the other population is currently more widespread, more offshore and more migratory, as found in bony fishes such as Atlantic cod (*Gadus morhua*)^66^ and European anchovy (*Engraulis encrasicolus*)^67^ and in marine mammals like the bottlenose dolphin (*Tursiops truncatus*)^68^. Intraspecific differences in movement and residency patterns is also increasingly being reported in sharks^69–71^, including in large predatory species as tiger sharks^70^. Importantly, our findings suggest that the abundance of the putative southerly population has declined significantly compared to pre-1990s levels. The apparent local depletion of tiger sharks at the south eastern distribution of the species in Australia supports previous studies showing a reduction in the abundance and mean size of tiger sharks caught in this region^23,24,46^. Although the species is not commonly commercially targeted off eastern Australia, it is caught as bycatch, in Queensland and New South Wales’ shark control programs and in recreational fisheries off New South Wales. Off Queensland, tiger shark catch-per-unit-effort has dropped by 74% over the past 25 years, while the average size declined by 21%^24^, with most of the decline occurring in the southern part of the state^23^. Catch-per-unit-effort also declined in the NSW beach meshing program^22^. This reported strong reduction in tiger shark abundance, which coincides with the increase in lethal shark mitigation measures, is of concern and may imply that ongoing lethal mitigation measures as the possible drivers of the observed population declines in the region. Recent estimates of tiger shark bycatch obtained from commercial logbook records indicate between 5 and 10 t routinely caught off Queensland, and approximately 3 t per year off New South Wales. However, New South Wales also accounts for approximately 10 t of tiger shark recreational catches^72,73^. Further, since 1936 the game fishing in New South Wales has targeted large tiger sharks for capture point scores and continues to do so over several competitions annually^71^. Illegal foreign fishing vessels that target large sharks for their fins have been apprehended in the Tasman Sea region and north into the Coral Sea, with tiger shark found to comprise about 20% of the total biomass of sharks caught^74^. Overall, these activities may have selectively removed the southern population (‘cluster 2’), most likely through asserting a higher level of exploitation than on the more widespread northern population. Ongoing climate change can also contribute to shifting distributions and abundance of marine fish species and populations^63^, particularly in Australia^75,76^, since the region has been identified as one of the global hotspots for ocean warming^77^. Thus, it is possible that increasing sea temperatures could have negatively affected the putative southern population or contributed to the increase of the northern population in southern waters. This ‘tropicalization’ of southern Australian waters is well studied with several ‘northern’ species shifting their distribution ranges further south, and increasing in abundance in the Tasman Sea (e.g.^78^). Nevertheless, in the light of the recent dramatic demographic changes in tiger sharks and other large shark populations in the region, it is likely that fishing has played a significant role in the reported changes in abundance of these two genetically differentiated populations. Still, genetic analysis of more contemporary samples from along the east coast of Australia and the Pacific Ocean^28^ using e.g. a targeted genotyping approach focusing on the most informative SNPs^79^, could help to further elucidate the apparent change of abundance of the two populations of tiger sharks in eastern Australian waters.

The apparent occurrence (and loss) of localized cryptic populations of tiger sharks, at a finer geographical level than previously believed, raises a number of concerns regarding identification and monitoring of intraspecific biodiversity in large sharks. Our study suggests that localized populations may be more common than anticipated from recent genetic studies using markers with lower resolution^28,80^. Thus, reported local, in particular coastal, depletions of large sharks^14^ could also be associated with severe reductions or losses of entire populations that has remained unnoticed due to replacement with individuals from other and possibly more wide-ranging populations. This eradication of semi-independent evolutionary lineages will not only have a local effect on population abundance, but also affect the evolutionary potential of the species as a whole and the ecosystem services they provide^4^. This highlights the importance of developing high-resolution genomic resources for elasmobranchs and other high gene flow marine organisms^81,82^ which can provide information on a large number of variable sites in the genome (e.g. SNPs). By genotyping many individuals for a high number of SNPs, it may be possible to identify putative populations in species with general low levels of genetic differentiation such as sharks. More importantly, our work also points to significant challenges regarding the scale of current management and biodiversity protection schemes for large sharks. Sustainable management of local populations, through matching the scale of governance with population structure, is important for the protection of the evolutionary legacy of the species and the potential for adapting to future environmental changes^83^. It is also important for the maintenance of healthy marine ecosystems that could provide services to human society^84^. Accordingly, management focus will need to include localized protection measures, such as local seasonal closures or marine reserves to properly match the geographic scale of the population^85^. Specifically, for the east coast of Australia, we recommend to further investigate our findings, to elucidate the current abundance and distribution of the two populations and establish measures to protect the putative southern population component, which appears to have faced a significant historical decline, primarily driven by either direct and indirect exploitation. Overall, our work highlights the need to include historical samples in studies of population structure of large migratory marine species, as these harbour a treasure trove of information and can shed light on complex demographic patterns and aid in the development of accurate conservation actions at relevant geographical scales.

## Methods

### Sample collection

Tiger shark specimens were caught over a time-span of close to 80 years (~1939–2015). Samples originated from north-eastern and eastern Australia, extending from the Gulf of Carpentaria (GCA), through the Coral Sea (CRS) to the Tasman Sea (TAS) (Fig. 1a). Contemporary tissue samples (2000–2015) were obtained as fin-clips from sharks caught in the Queensland Shark Control Program, the New South Wales Shark Meshing Program, commercial and recreational landings, and sharks caught for tagging and tracking research purposes^86^. Historical samples (~1939–1999) comprised dried tiger shark jaws and vertebrae obtained from museum collections, fishers, and other private or public collections. The initial dataset consisted of 115 unique sharks. As tiger sharks are long-lived and the sampled individuals were highly variable in age, we estimated the year of birth for each sample to allow for a more accurate temporal genetic comparison (Fig. 1b). For example, a large shark sampled in 2010 could have been born the same year as a smaller shark sampled in 1990. Individual year of birth was estimated using a locally derived relationship between total length (L_T_) and age. Growth rate estimation (t) was based on vertebral aging for both females and males using the Von Bertalanffy growth function (VBGF) (1) as^76^:

1 $$ {\text{t }} = {\text{ ln }}\left( {(( - {\text{L}}_{{\text{T}}} + {\text{ L}}_{\infty } )/({\text{L}}_{\infty } - {\text{L}}_{0} ))/ - {\text{k}}} \right), $$

where L_0_ and L_∞_ represent the length-at-birth and theoretical asymptotic length, respectively, and k represents the growth coefficient. We assumed different parameters for males and females (males: L_∞_ = 441.1 cm, k = 0.08, and L_0_ = 123.4 cm; females: L_∞_ = 379.9 cm, k = 0.06, and L_0_ = 116.8 cm), and a combined set where information on sex was not available (L_∞_ = 433.7 cm, k = 0.06, and L_0_ = 121.5 cm). For individuals with only fork length (L_F_) available, total length was calculated using the relationship L_T_ = 22.607 + 1.096 L_F_^87^. For the 20 sharks without length information, total weight (W_T_) was used to first obtain L_F_ using a regression equation parameterized for tiger sharks in the north-western Atlantic^88^, the closest population to our target species from which there is available data (L_F_ = ((W_T_/2.5281) × 10^–6^)^(1/3.2603)^).

### DNA extraction and target capture

Historical tissue material was collected following the protocol described in Nielsen et al.^39^ and involved the collection of “bio-swarf” produced when drilling a 3.5-mm hole in the calcified cartilage of jaws or vertebrae. Extraction of DNA from the bio-swarf and contemporary fin tissue was performed with the Bioline ISOLATE II Genomic DNA kit according to the manufacturer’s protocol, using 18–37 mg (average 27 mg) of tissue per extraction. For genomic library preparation, DNA from contemporary samples was sheared to an average fragment size of 200 bp with a M220 focused ultrasonicator (Covaris, USA). DNA from historical material was fragmented due to degradation over time and therefore used directly. Genomic-capture libraries were prepared using the KAPA Hyper Prep Kit (Kapa Biosystems, USA) according to manufacturer’s instructions. A total of 50 ng input DNA per sample was used with a one in five dilution of the TruSeq DNA HT dual-index adaptors (Illumina, USA). Ten PCR cycles for library amplification were used for the contemporary samples and twelve for the historical. Selected regions of genomic DNA were captured with a MyBaits (MYcroarray) target enrichment kit, consisting of 20.000 biotinylated RNA baits (120 bp each) developed from pancreas, liver and brain derived transcriptome sequences^89^ of the small-spotted catshark (*Scyliorhinus canicula*). At the time of bait development this was the most taxonomically similar species from which a large genomic resource was available for bait design that would likely capture tiger shark transcribed regions. For more details about DNA extraction, library preparation, and bait design see Nielsen et al.^39^. All DNA samples were captured individually, using 135 ng of DNA library as input, which had previously been treated with a 1× AMPure XP beads clean-up. Hybridization capture of tiger shark DNA was conducted for 24 h at 60 °C in solution for subsequent paired-end (2 × 125 bp) sequencing on an Illumina HiSeq2000 v4. Prior to sequencing, the captured libraries were amplified using thirteen PCR cycles and purified using 0.8× AMPure XP beads. Quality Control (QC) steps were performed using a Bioanalyzer (Agilent Technologies, CA, USA), thus the final sequencing libraries could be pooled in equal nM concentrations. Samples were sequenced in two lanes. One “historical” lane consisting of 38 jaw samples and 5 fin samples, and a “contemporary” lane with 75 fin samples and 2 jaw samples. The difference in sample number per lane accounted for more variable template numbers among historical samples and thus secured a higher minimum number of sequences per individual. The lanes reciprocally included the same jaw samples that were sequenced in both lanes, similar to one of the contemporary tissue samples in the historical lane, allowing for estimation of “lane effects” and to evaluate reproducibility of multilocus genotypes through the molecular, bioinformatics and population genomics pipelines.

### Bioinformatics pipeline and data filtering

We customized a bioinformatics pipeline to ensure removal of potential contaminants, artefacts and low quality reads^90^ before proceeding with the downstream analysis. Briefly, the de-multiplexed reads were controlled for quality using FastQC^91^. All adaptors were removed using AdapterRemoval^92^ and reads were filtered by length and quality, with a minimum length of 30 bp and base quality of 28. Filtered reads were merged using FLASH^93^ with default parameters, and checked for contaminants using Kraken2^94^. Both unpaired and concordantly merged reads were mapped against possible sources of contaminants (bacteria, fungi) using the Bowtie2^95^ “sensitive” option. The cleaned reads were also mapped against the mitochondrial genome of tiger shark (NCBI Reference Sequence: NC_022193.1^96^). Previous studies have shown that “off-target capture” is common, i.e. capture of genomic regions of both nuclear and mitochondrial origin not matching the baits. For example, in highly degraded samples target template DNA may not bind and amplify as well as in good quality samples and templates, resulting in more amplification of non-targeted regions of the genome^10,11^. Moreover, mtDNA sequences are commonly captured, or directly sequenced, due to the high copy number of mitochondrial DNA compared to nuclear DNA^82,97^. This phenomenon may even be desirable, as it allows assessment of mtDNA diversity. After removal of mitochondrial sequences, the reads were mapped against the transcriptome of tiger shark^45^, using the BWA-mem algorithm. This transcriptome includes 179,867 unique contigs greater than200 bp length (average 822 bp, maximum 15,892 bp). After mapping, PCR duplicates were removed using Picard-tools (http://broadinstitute.github.io/picard/). We checked the patterns of DNA damage of the remaining reads, using Mapdamage2.0^98^. Coverage and depth of the target regions were estimated using Samtools^99^, and finally we called SNPs using Freebayes^100^ with default parameters. The raw SNPs obtained were further filtered to keep only biallelic SNPs with quality above 30 and minimum allele count of three. Only SNPs with a maximum level of missing data of 20% were maintained. Additionally, we filtered for excess depth to reduce the possible presence of paralogs and multi-copy loci. Linkage disequilibrium (LD) between SNPs within bins of 800 bp (maximum length of the merged reads plus 150 bp each side) was estimated using the prune function in bcftools (SAMtools package), by calculating the square correlation between alleles of each pair of loci, r^2^^101^ and keeping only SNPs with an r^2^ < 0.25. To test the reliability of our final SNPs, we compared genotypes for duplicate control samples and only maintained SNPs that matched more than 80% of the pairwise comparisons (to allow for missing data). Finally, we filtered for significant departure from HWE (*p* < 0.05) to remove systematic genotyping errors. All filtering steps but the LD pruning were done using VCFtools^102^.

### Data analysis for temporal and spatial genomic variability

Back-calculated year of birth ranged between 1917 (the oldest) and 2012 (the youngest). For all downstream analysis, we grouped samples into four time periods based on their estimated decade of birth: 1910–1960, 1970–1990 and 2000, the latter comprising all contemporary samples (2000–2015). These date ranges were used as named time periods throughout the manuscript. The three periods were also associated with different catch rates in the study area, with the highest catch rate between 1960 and 1980, which significantly decreased after 2000^24^, especially in the southern part of Queensland^9,103^. For the temporal analysis we estimated temporal genetic differentiation using all samples (n = 106) including individuals belonging to different spatiotemporal sample groups, namely GCA_1970-1990 (n = 12), GCA_2000 (n = 14), CRS_1970-1990 (n = 7), CRS_2000 (n = 15), TAS_1910-1960 (n = 19), TAS_1970-1990 (n = 14) and TAS_2000 (n = 26). All estimations of pairwise F_ST_^104^ between spatial and temporal samples were performed using the R package *StAMPP*^105^ and their significance was assessed with 1,000 permutations over loci. For all FST comparisons the p-values were adjusted using a False Discovery Rate (FDR) method. A Principal Coordinates Analysis (PCoA) was applied to the pairwise F_ST_ matrix to summarize and plot the differences reported in the table using the pcoa function in the *ape* 5.0 package^106^ in R. We performed a Principal Component Analysis (PCA) to explore the spatial and temporal structure of individual genotypes using the R package *adegenet*^107^. An initial PCA of all samples revealed two identical genotypes (two types of archived tissue from the same individual) and one of them was subsequently removed. A PCA of the contemporary samples revealed two extreme outliers of which one of them was identified as another species (spinner shark; *Carcharhinus brevipinna*) based on mtDNA sequences. Both samples were removed from further analysis. For the temporal estimates the dataset (n = 106) was divided into the three periods: 1910–1960 (n = 19), 1970–1990 (n = 33) and 2000 (n = 55). In order to assess current spatial genetic differentiation a subset of 74 contemporary individuals (based on year of catch) from the three different areas: GCA (n = 26), CRS (n = 21) and TAS (n = 27) were selected. In order to test for possible differences in individual heterozygosity among population groups, we used an ad-hoc R script, to calculate the proportion of heterozygous loci out of all genotyped loci for an individual, thereby accounting for differences in total number of genotyped loci (i.e. missing data) across individuals. The boxplot was realized using the *ggplot2* package in R^108^ to highlight the median of each cluster. The distribution of F_ST_ across loci was plotted as a function of heterozygosity using ggplot2. Estimates of Weir and Cockerham^104^ F_ST_ and heterozygosity were obtained using VCFtools. A test for selection was performed using *pcadapt*^109^, with a qvalue of 0.1 as cut-off to identify putative selective outliers and bayescan^99^ with parameters ‐n 5000 ‐thin 10 ‐nbp 20 ‐pilot 5000 ‐burn 50000 ‐pr_odds 100. To detect the presence of possible genetic clusters identified by the pairwise F_ST_ analysis, a Discriminant Analysis of Principal Components (DAPC) was applied as it better detects variability and division among populations compared to other software for population structure analysis (e.g. STRUCTURE^110^) as it is free of assumptions about any underlying population genetics model. For the DAPC we used the *adegenet* package in R. Since the results of a DAPC analysis can be highly affected by the number of discriminant functions used, we ran the *xvalDapc* function first to select the best number of discriminant functions to represent the data. The model was corrected for overfitting by using the a-score estimate, with 26 PCs retained representing the optimal trade-off between power of discrimination and over-fitting. The recommended number of PCs obtained using this approach (26) was used to run a Bayesian information criterion (BIC) analysis and the k-means algorithm to identify the most likely number of clusters that could explain the variability within our dataset. For comparison, the *snmf* function implemented in the LEA package^111^ was also tested, since it represents a different model-based approach for clusters identification in large datasets. The value of K that best explain the data was selected using the cross-entropy criterion, with 100 repetitions for K value. A Fishers exact test was applied to pairwise comparisons between different temporal collections to determine whether any possible change in relative proportion among groups was statistically significant. Estimates of relatedness were performed using VCFtools to exclude kin sampling that could skew the estimates of genetic diversity. Finally, Site Frequency Spectrum per temporal and spatial collections were generated in ANGSD^112^ to further investigate whether our results could be affected by technical biases (e.g. excess of singletons). Due to the lack of knowledge on the ancestral state, the folded version of the “realSFS” function was used to polarize the allele frequencies on the reference used to produce the vcf file (-anc -fold 1). In addition, Pi and Watterson theta were estimated for each temporal and spatial group using the “sfsr” function (https://github.com/andrewparkermorgan/sfsr).

## Supplementary Information

Supplementary Information.
