# Supplementary material for: Retrospective genomics highlights changes in genetic composition of tiger sharks (Galeocerdo cuvier) and potential loss of a south-eastern Australia population
Source: Sci Rep. 2022 Apr 21;12:6582. doi: 10.1038/s41598-022-10529-w (PMC9023511; doi:10.1038/s41598-022-10529-w)

**Supplementary materials**

**Table S1. Final dataset for the 106 samples included in the analyses.** The table reports in the following order from left to right: sample ID (ID_tube), location of catch (Location), region (Region, CRS = Coral Sea, GCA = Gulf of Carpentaria, TAS = Tasman Sea), estimated year of birth (Birth Year) and temporal period to which they were assigned (Decade Birth), approximate year of catch (Catch Year) and consequent temporal period assigned(Decade Catch), estimated age of sample (Age) and assigned cluster after DAPC analysis (Cluster). The symbol “*” refers to samples for which we don’t have an exact estimate of birth, but rather general information (a=adult, b=caught in 1962, thus born in the “historical group”).

| **ID_tube** | **Location** | **Region** | **Birth Year** | **Decade Birth** | **Catch Year** | **Decade Catch** | **Group** | **Age** | **TL (cm)** | **Cluster** |
| --- | --- | --- | --- | --- | --- | --- | --- | --- | --- | --- |
| 5_421P | Northern Territory | GCA | 1993 | 1970-1990 | 2008 | 2000-2010 | GCA_1970-90 | 15 | 273.0 | 1 |
| 5_422P | Northern Territory | GCA | 1990 | 1970-1990 | 2008 | 2000-2010 | GCA_1970-90 | 18 | 290.0 | 1 |
| 5_423P | Northern Territory | GCA | 1997 | 1970-1990 | 2008 | 2000-2010 | GCA_1970-90 | 11 | 246.0 | 1 |
| 5_425P | Northern Territory | GCA | 1994 | 1970-1990 | 2008 | 2000-2010 | GCA_1970-90 | 14 | 335.0 | 1 |
| 5_426P | Northern Territory | GCA | 1999 | 1970-1990 | 2008 | 2000-2010 | GCA_1970-90 | 9 | 289.0 | 1 |
| 5_431P | Northern Territory | GCA | 1995 | 1970-1990 | 2008 | 2000-2010 | GCA_1970-90 | 13 | 262.0 | 1 |
| 5_433P | Northern Territory | GCA | 1989 | 1970-1990 | 2008 | 2000-2010 | GCA_1970-90 | 19 | 296.0 | 1 |
| 5_434P | Northern Territory | GCA | 1998 | 1970-1990 | 2008 | 2000-2010 | GCA_1970-90 | 10 | 239.0 | 1 |
| 5_445P | Northern Territory | GCA | 1977 | 1970-1990 | 2012 | 2000-2010 | GCA_1970-90 | 35 | 350.0 | 1 |
| 5_446P | Northern Territory | GCA | 1989 | 1970-1990 | 2012 | 2000-2010 | GCA_1970-90 | 23 | 314.0 | 1 |
| 5_448P | Northern Territory | GCA | 1999 | 1970-1990 | 2012 | 2000-2010 | GCA_1970-90 | 13 | 261.0 | 1 |
| 5_451P | Northern Territory | GCA | 1997 | 1970-1990 | 2008 | 2000-2010 | GCA_1970-90 | 11 | 244.0 | 1 |
| 5_417P | Northern Territory | GCA | 2004 | 2000 | 2008 | 2000-2010 | GCA_2000 | 4 | 214.0 | 1 |
| 5_418P | Northern Territory | GCA | 2003 | 2000 | 2008 | 2000-2010 | GCA_2000 | 5 | 222.0 | 1 |
| 5_419P | Northern Territory | GCA | 2002 | 2000 | 2008 | 2000-2010 | GCA_2000 | 6 | 250.0 | 1 |
| 5_424P | Northern Territory | GCA | 2004 | 2000 | 2008 | 2000-2010 | GCA_2000 | 4 | 205.0 | 1 |
| 5_427P | Northern Territory | GCA | 2003 | 2000 | 2008 | 2000-2010 | GCA_2000 | 5 | 230.0 | 1 |
| 5_429P | Northern Territory | GCA | 2001 | 2000 | 2008 | 2000-2010 | GCA_2000 | 7 | 253.0 | 2 |
| 5_430P | Northern Territory | GCA | 2000 | 2000 | 2008 | 2000-2010 | GCA_2000 | 8 | 217.0 | 1 |
| 5_432P | Northern Territory | GCA | 2005 | 2000 | 2008 | 2000-2010 | GCA_2000 | 3 | 200.0 | 1 |
| 5_435P | Northern Territory | GCA | 2001 | 2000 | 2008 | 2000-2010 | GCA_2000 | 7 | 256.0 | 1 |
| 5_436P | Northern Territory | GCA | 2003 | 2000 | 2008 | 2000-2010 | GCA_2000 | 5 | 233.0 | 1 |
| 5_444P | Northern Territory | GCA | 2000 | 2000 | 2009 | 2000-2010 | GCA_2000 | 9 | 229.0 | 1 |
| 5_449P | Northern Territory | GCA | 2003 | 2000 | 2012 | 2000-2010 | GCA_2000 | 9 | 289.0 | 1 |
| 5_450P | Northern Territory | GCA | 2012 | 2000 | 2012 | 2000-2010 | GCA_2000 | 0 | 80.0 | 1 |
| 5_452P | Northern Territory | GCA | 2003 | 2000 | 2008 | 2000-2010 | GCA_2000 | 5 | 221.0 | 1 |
| 5_101P | Mackay | CRS | 1976 | 1970-1990 | 2011 | 2000-2010 | CRS_1970-90 | 35 | 390.0 | 1 |
| 5_90P | Mackay | CRS | 1998 | 1970-1990 | 2011 | 2000-2010 | CRS_1970-90 | 13 | 258.0 | 1 |
| 5_93P | Mackay | CRS | 1996 | 1970-1990 | 2011 | 2000-2010 | CRS_1970-90 | 15 | 276.0 | 1 |
| 5_c5P | Cairns | CRS | 1980 | 1970-1990 | 2015 | 2000-2010 | CRS_1970-90 | 35 | 360.0 | 1 |
| 5_c6P | Cairns | CRS | 1991 | 1970-1990 | 2015 | 2000-2010 | CRS_1970-90 | 24 | 317.0 | 1 |
| 5_c7P | Cairns | CRS | 1997 | 1970-1990 | 2015 | 2000-2010 | CRS_1970-90 | 18 | 292.0 | 1 |
| 5_c8P | Cairns | CRS | 1990 | 1970-1990 | 2015 | 2000-2010 | CRS_1970-90 | 25 | 322.0 | 1 |
| 5_102P | Mackay | CRS | 2008 | 2000 | 2011 | 2000-2010 | CRS_2000 | 3 | 200.0 | 1 |
| 5_10P | Mackay | CRS | 2002 | 2000 | 2011 | 2000-2010 | CRS_2000 | 9 | 226.0 | 1 |
| 5_442P | North Queensland | CRS | 2008 | 2000 | 2008 | 2000-2010 | CRS_2000 | 0 | 113.0 | 1 |
| 5_443P | North Queensland | CRS | 2007 | 2000 | 2008 | 2000-2010 | CRS_2000 | 1 | 126.0 | 1 |
| 5_87P | Mackay | CRS | 2005 | 2000 | 2011 | 2000-2010 | CRS_2000 | 6 | 196.0 | 1 |
| 5_88P | Mackay | CRS | 2011 | 2000 | 2011 | 2000-2010 | CRS_2000 | 0 | 111.0 | 1 |
| 5_89P | Mackay | CRS | 2009 | 2000 | 2011 | 2000-2010 | CRS_2000 | 2 | 165.0 | 1 |
| 5_91P | Mackay | CRS | 2003 | 2000 | 2011 | 2000-2010 | CRS_2000 | 8 | 216.3 | 1 |
| 5_92P | Mackay | CRS | 2002 | 2000 | 2011 | 2000-2010 | CRS_2000 | 9 | 227.0 | 1 |
| 5_96P | Mackay | CRS | 2005 | 2000 | 2011 | 2000-2010 | CRS_2000 | 6 | 194.0 | 2 |
| 5_98P | Mackay | CRS | 2005 | 2000 | 2011 | 2000-2010 | CRS_2000 | 6 | 242.0 | 1 |
| 5_99P | Mackay | CRS | 2007 | 2000 | 2011 | 2000-2010 | CRS_2000 | 4 | 208.0 | 1 |
| 5_c4P | Cairns | CRS | 2008 | 2000 | 2015 | 2000-2010 | CRS_2000 | 7 | 210.0 | 1 |
| 5_c9P | Cairns | CRS | 2010 | 2000 | 2015 | 2000-2010 | CRS_2000 | 5 | 228.0 | 1 |
| 6_OTS14P | Lake Macquarie | TAS | 1956 | 1910-1960 | 1979 | 1970-1990 | TAS_1910-1960 | 23 | 353.3 | 2 |
| 6_OTS20P | Lake Macquarie | TAS | 1950 | 1910-1960 | 1981 | 1970-1990 | TAS_1910-1960 | 31 | 384.4 | 2 |
| 6_OTS21P | Lake Macquarie | TAS | 1966 | 1910-1960 | 1985 | 1970-1990 | TAS_1910-1960 | 19 | 333.9 | 2 |
| 6_OTS22P | Lake Macquarie | TAS | 1956 | 1910-1960 | 1985 | 1970-1990 | TAS_1910-1960 | 29 | 379.6 | 2 |
| 6_OTS23P | Port Stephens | TAS | 1956 | 1910-1960 | 1985 | 1970-1990 | TAS_1910-1960 | 29 | 377.9 | 1 |
| 6_OTS24P__CTG_ | Lake Macquarie | TAS | ~1940-50s | 1910-1960 | 1970-80s | 1970-1990 | TAS_1910-1960 | 35 | 398.0 | 2 |
| 6_OTS25P | Lake Macquarie | TAS | 1953 | 1910-1960 | 1978 | 1970-1990 | TAS_1910-1960 | 25 | 365.1 | 2 |
| 6_OTS26P__CGG_ | Broken Bay | TAS | 1950 | 1910-1960 | 1985 | 1970-1990 | TAS_1910-1960 | 35 | 408.1 | 2 |
| 6_OTS28P | Broken Bay | TAS | 1968 | 1910-1960 | 1997 | 1970-1990 | TAS_1910-1960 | 29 | 379.1 | 2 |
| 6_OTS29P | Broken Bay | TAS | 1963 | 1910-1960 | 1996 | 1970-1990 | TAS_1910-1960 | 33 | 390.0 | 1 |
| 6_OTS30P | Lake Macquarie | TAS | 1957 | 1910-1960 | 1986 | 1970-1990 | TAS_1910-1960 | 29 | 379.6 | 1 |
| 6_OTS31P | Sydney | TAS | ~1947 | 1910-1960 | ~1966 | 1910-1960 | TAS_1910-1960 | 19 | 333.1 | 1 |
| 6_OTS32P | Bondi Beach | TAS | ~1920 | 1910-1960 | late 1930 | 1910-1960 | TAS_1910-1960 | 16-19 | 321.6 - 333.1 | 1 |
| 6_OTS33P | Bermagui | TAS | 1917 | 1910-1960 | 1952 | 1910-1960 | TAS_1910-1960 | 35 | 409.3 | 2 |
| 6_OTS34P__GAG_ | Botany Bay | TAS | < 1960s | 1910-1960 | 1962 | 1910-1960 | TAS_1910-1960 | *^b^ | * ^b^ | 1 |
| 6_OTS38P | Southport | TAS | ~1965 | 1910-1960 | 1980 | 1970-1990 | TAS_1910-1960 | ~15*^a^ | >300*^a^ | 2 |
| 6_OTS3P | Broken Bay | TAS | 1969 | 1910-1960 | 1980 | 1970-1990 | TAS_1910-1960 | 11 | 272.8 | 2 |
| 6_OTS42P | Botany Bay | TAS | ~1965 | 1910-1960 | 1980 | 1970-1990 | TAS_1910-1960 | ~15*^a^ | >300*^a^ | 2 |
| 6_OTS43P | Botany Bay | TAS | ~1969 | 1910-1960 | 1984 | 1970-1990 | TAS_1910-1960 | ~15*^a^ | >300*^a^ | 2 |
| 5_323P | Port Stephens | TAS | 1995 | 1970-1990 | 2009 | 1970-1990 | TAS_1970-90 | 14 | 268.0 | 1 |
| 6_OTS10P | Bermagui | TAS | ~1970 | 1970-1990 | 1985 | 1970-1990 | TAS_1970-90 | ~15*^a^ | >300*^a^ | 2 |
| 6_OTS11P | Bermagui | TAS | ~1981 | 1970-1990 | 1996 | 1970-1990 | TAS_1970-90 | ~15*^a^ | >300*^a^ | 2 |
| 6_OTS12P | Bermagui | TAS | ~1978 | 1970-1990 | 1993 | 1970-1990 | TAS_1970-90 | ~15*^a^ | >300*^a^ | 1 |
| 6_OTS41P | Botany Bay | TAS | 1972 | 1970-1990 | 1980 | 1970-1990 | TAS_1970-90 | 8 | 243.0 | 1 |
| 6_OTS44P | Seal Rocks (PS) | TAS | 1975 | 1970-1990 | 1988 | 1970-1990 | TAS_1970-90 | 13 | 292.4 | 1 |
| 6_OTS45P | Botany Bay | TAS | 1976 | 1970-1990 | 1980 | 1970-1990 | TAS_1970-90 | 4 | 194.0 | 2 |
| 6_OTS46P | Botany Bay | TAS | 1972 | 1970-1990 | 1980 | 1970-1990 | TAS_1970-90 | 8 | 240.0 | 2 |
| 6_OTS47P | Botany Bay | TAS | 1971 | 1970-1990 | 1980 | 1970-1990 | TAS_1970-90 | 9 | 254.0 | 2 |
| 6_OTS5P | Bermagui | TAS | ~1973 | 1970-1990 | 1988 | 1970-1990 | TAS_1970-90 | ~15*^a^ | >300*^a^ | 1 |
| 6_OTS6P | Bermagui | TAS | ~1973 | 1970-1990 | 1988 | 1970-1990 | TAS_1970-90 | ~15*^a^ | >300*^a^ | 1 |
| 6_OTS7P | Bermagui | TAS | ~1973 | 1970-1990 | 1988 | 1970-1990 | TAS_1970-90 | ~15*^a^ | >300*^a^ | 1 |
| 6_OTS8P | Bermagui | TAS | ~1975 | 1970-1990 | 1990 | 1970-1990 | TAS_1970-90 | ~15*^a^ | >300*^a^ | 1 |
| 6_OTS9P | Mallacoota | TAS | 1975 | 1970-1990 | 1989 | 1970-1990 | TAS_1970-90 | 14 | 301.4 | 1 |
| 5_223P | Port Macquarie | TAS | 2001 | 2000 | 2007 | 2000-2010 | TAS_2000 | 6 | 196.0 | 1 |
| 5_225P | Coffs Harbour | TAS | 2007 | 2000 | 2008 | 2000-2010 | TAS_2000 | 1 | 140.0 | 1 |
| 5_231P | Tweed Heads | TAS | 2006 | 2000 | 2009 | 2000-2010 | TAS_2000 | 3 | 163.0 | 1 |
| 5_232P | Tweed Heads | TAS | 2005 | 2000 | 2009 | 2000-2010 | TAS_2000 | 4 | 167.0 | 1 |
| 5_233P | Port Macquarie | TAS | 2006 | 2000 | 2009 | 2000-2010 | TAS_2000 | 3 | 155.0 | 1 |
| 5_235P | Port Macquarie | TAS | 2009 | 2000 | 2009 | 2000-2010 | TAS_2000 | 0 | 120.0 | 1 |
| 5_236P | Port Macquarie | TAS | 2006 | 2000 | 2009 | 2000-2010 | TAS_2000 | 3 | 182.0 | 1 |
| 5_237P | Port Macquarie | TAS | 2006 | 2000 | 2009 | 2000-2010 | TAS_2000 | 3 | 165.0 | 1 |
| 5_238P | Tweed Heads | TAS | 2008 | 2000 | 2009 | 2000-2010 | TAS_2000 | 1 | 157.0 | 1 |
| 5_239P | Tweed Heads | TAS | 2006 | 2000 | 2009 | 2000-2010 | TAS_2000 | 3 | 158.0 | 1 |
| 5_241P | Coffs Harbour | TAS | 2007 | 2000 | 2009 | 2000-2010 | TAS_2000 | 2 | 160.0 | 1 |
| 5_242P | Coffs Harbour | TAS | 2005 | 2000 | 2009 | 2000-2010 | TAS_2000 | 4 | 216.0 | 1 |
| 5_245P | Evans Head | TAS | 2008 | 2000 | 2009 | 2000-2010 | TAS_2000 | 1 | 139.0 | 1 |
| 5_251P | Coffs Harbour | TAS | 2009 | 2000 | 2009 | 2000-2010 | TAS_2000 | 0 | 117.0 | 1 |
| 5_252P | Evans Head | TAS | 2009 | 2000 | 2009 | 2000-2010 | TAS_2000 | 0 | 128.0 | 1 |
| 5_253P | Evans Head | TAS | 2007 | 2000 | 2009 | 2000-2010 | TAS_2000 | 2 | 169.0 | 1 |
| 5_255P | Tweed Heads | TAS | 2009 | 2000 | 2009 | 2000-2010 | TAS_2000 | 0 | 117.0 | 1 |
| 5_311P | Port Stephens | TAS | 2000 | 2000 | 2007 | 2000-2010 | TAS_2000 | 7 | 260.0 | 1 |
| 5_326P | Port Stephens | TAS | 2001 | 2000 | 2009 | 2000-2010 | TAS_2000 | 8 | 280.0 | 1 |
| 5_327P | Port Stephens | TAS | 2002 | 2000 | 2009 | 2000-2010 | TAS_2000 | 7 | 256.0 | 1 |
| 5_331P | Port Stephens | TAS | 2003 | 2000 | 2010 | 2000-2010 | TAS_2000 | 7 | 265.0 | 1 |
| 5_339P | Port Stephens | TAS | 2004 | 2000 | 2011 | 2000-2010 | TAS_2000 | 7 | 265.0 | 1 |
| 5_343P | Port Stephens | TAS | 2005 | 2000 | 2011 | 2000-2010 | TAS_2000 | 6 | 251.0 | 1 |
| 6_234P_P | Port Macquarie | TAS | 2006 | 2000 | 2009 | 2000-2010 | TAS_2000 | 3 | 160.0 | 1 |
| 6_240P_P | Tweed Heads | TAS | 2008 | 2000 | 2009 | 2000-2010 | TAS_2000 | 1 | 148.0 | 1 |
| 6_254P_P | Evans Head | TAS | 2005 | 2000 | 2009 | 2000-2010 | TAS_2000 | 4 | 171.0 | 1 |

**Table S2. Sample sizes for the spatiotemporal age back-calculated samples.** Sample sizes below six were not included in estimates of temporal differentiation (F_ST_). Region codes as per Table S1.

| Year of birth/Location | Gulf of Carpentaria (GCA) | Coral Sea  (CRS) | Tasman Sea  (TAS) |
| --- | --- | --- | --- |
| 1910-1960 | 0 | 0 | 19 |
| 1970-1990 | 12 | 7 | 14 |
| 2000 | 14 | 14 | 26 |
| **Total per location** | 26 | 21 | 59 |

**Table S3**. **Pairwise F_ST_ estimates among all collection.** Pairwise F_ST_ estimates (lower diagonal) and p-values (upper diagonal) between all collection. Sample size per collection: GCA_1970–1990 (12), GCA_2000 (14), CRS_1970–1990 (7), CRS_2000 (14), TAS_1910–1960 (19), TAS_1970–1990 (14), TAS_2000 (26). *significant comparisons (p-values < 0.05).

|  | **GCA_1970–90** | **GCA_2000** | **CRS_1970–90** | **CRS_2000** | **TAS_1910–1960** | **TAS_1970–90** | **TAS_2000** |
| --- | --- | --- | --- | --- | --- | --- | --- |
| **GCA_1970–90** | - | 0.779 | 0.698 | 0.449 | 0.000* | 0.165 | 0.632 |
| **GCA_2000** | 0.000 | - | 0.693 | 0.342 | 0.000* | 0.033* | 0.650 |
| **CRS_1970–90** | 0.000 | 0.000 | - | 0.752 | 0.000* | 0.088 | 0.459 |
| **CRS_2000** | 0.000 | 0.006 | 0.000 | - | 0.000* | 0.010* | 0.102 |
| **TAS_1910–1960** | 0.005 | 0.002 | 0.006 | 0.006 | - | 0.357 | 0.000* |
| **TAS_1970–90** | 0.001 | 0.000 | 0.002 | 0.003 | 0.000 | - | 0.000* |
| **TAS_2000** | 0.000 | 0.000 | 0.000 | 0.001 | 0.006 | 0.003 | - |

**Supplementary Table S4**: Estimates of Theta Watterson (Theta W) and Theta Pi for each location per temporal period. Names of the locations correspond to Table S1.

| **Region** | **TAS** | **TAS** | **TAS** | **CRS** | **CRS** | **GCA** | **GCA** |
| --- | --- | --- | --- | --- | --- | --- | --- |
| **Temporal period** | 1910-1960 | 1970-1990 | 2000 | 1970-1990 | 2000 | 1970-1990 | 2000 |
| **Sample size** | 19 | 14 | 26 | 7 | 14 | 12 | 14 |
| **Theta W** | 0.045 | 0.045 | 0.052 | 0.064 | 0.049 | 0.057 | 0.064 |
| **Theta Pi** | 0.025 | 0.028 | 0.026 | 0.048 | 0.030 | 0.036 | 0.038 |

**Supplementary figures**

**Fig. S1. Discriminant Analysis of Principal Components (DAPC) to detect best number of clusters K (K = 2).**

The plots illustrate the value of BIC value for different clusters number (K 1 to 10), using **a.** the DAPC k-means method and **b.** the cross-entropy method implemented in the LEA package.

**
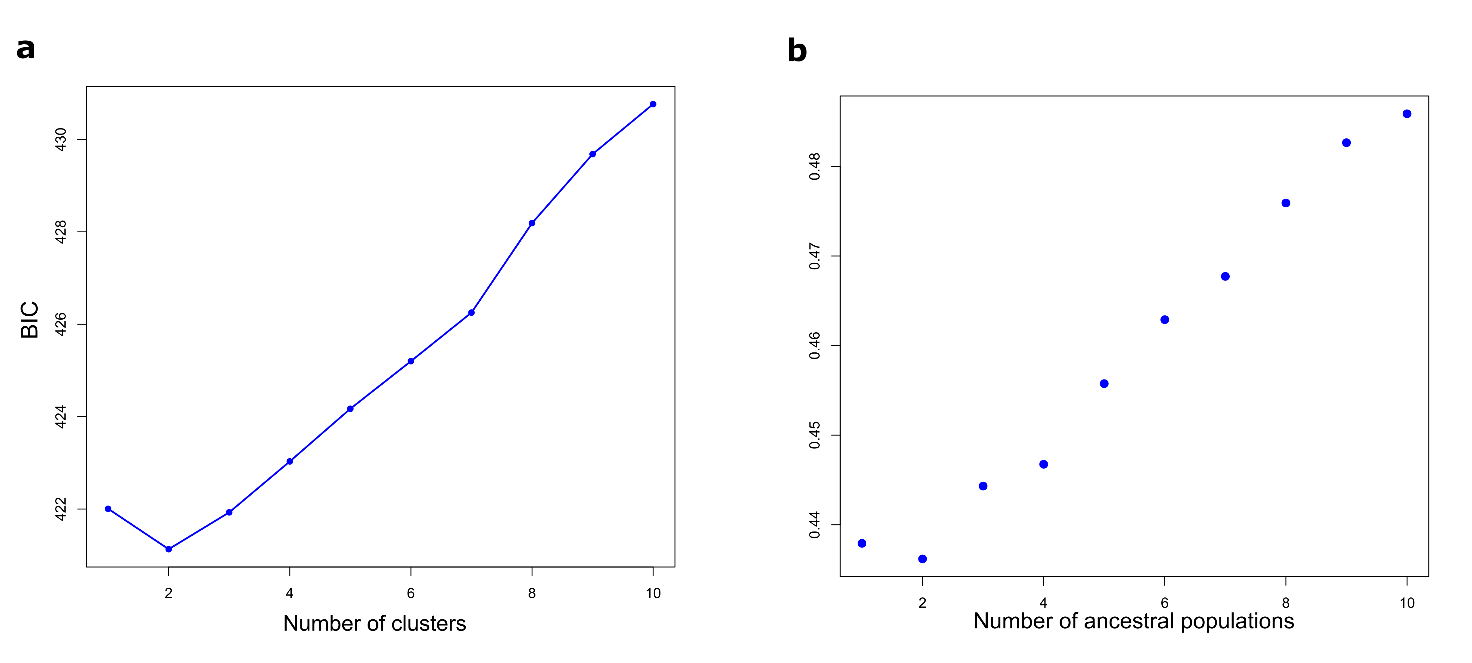
**

**Supplementary Figure S2: Site Frequency Spectrum (SFS) for each location per temporal period.** Each plot report the SFS estimated for each spatial and temporal collection. Groups names correspond to those in Table S1. Samples sizes are reported in Table S2.


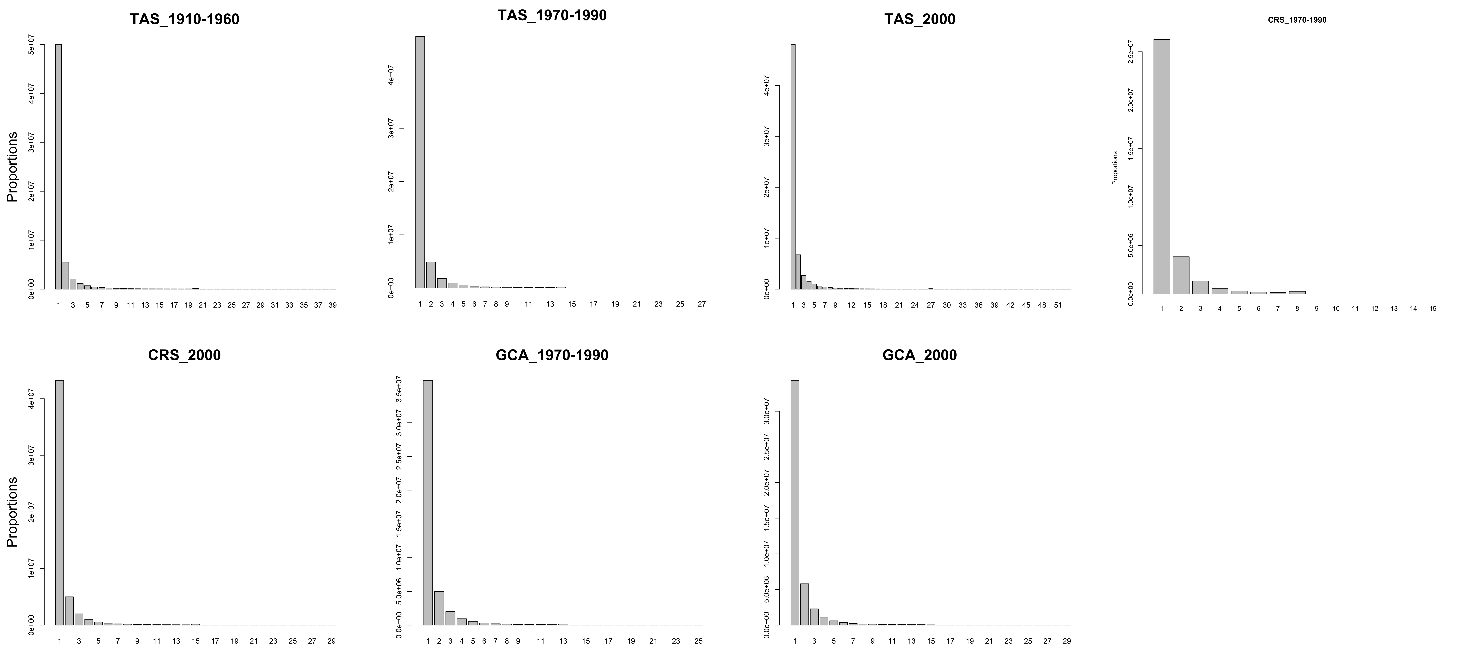


**Fig. S3. Distribution of F_ST_ as a function of heterozygosity.** Distribution of F_ST_ (y-axis) across 1,840 SNP loci between the two clusters (cluster ‘1’ and ‘2’) as a function of heterozygosity, reported on the x-axis. Each dot represents a single SNP.


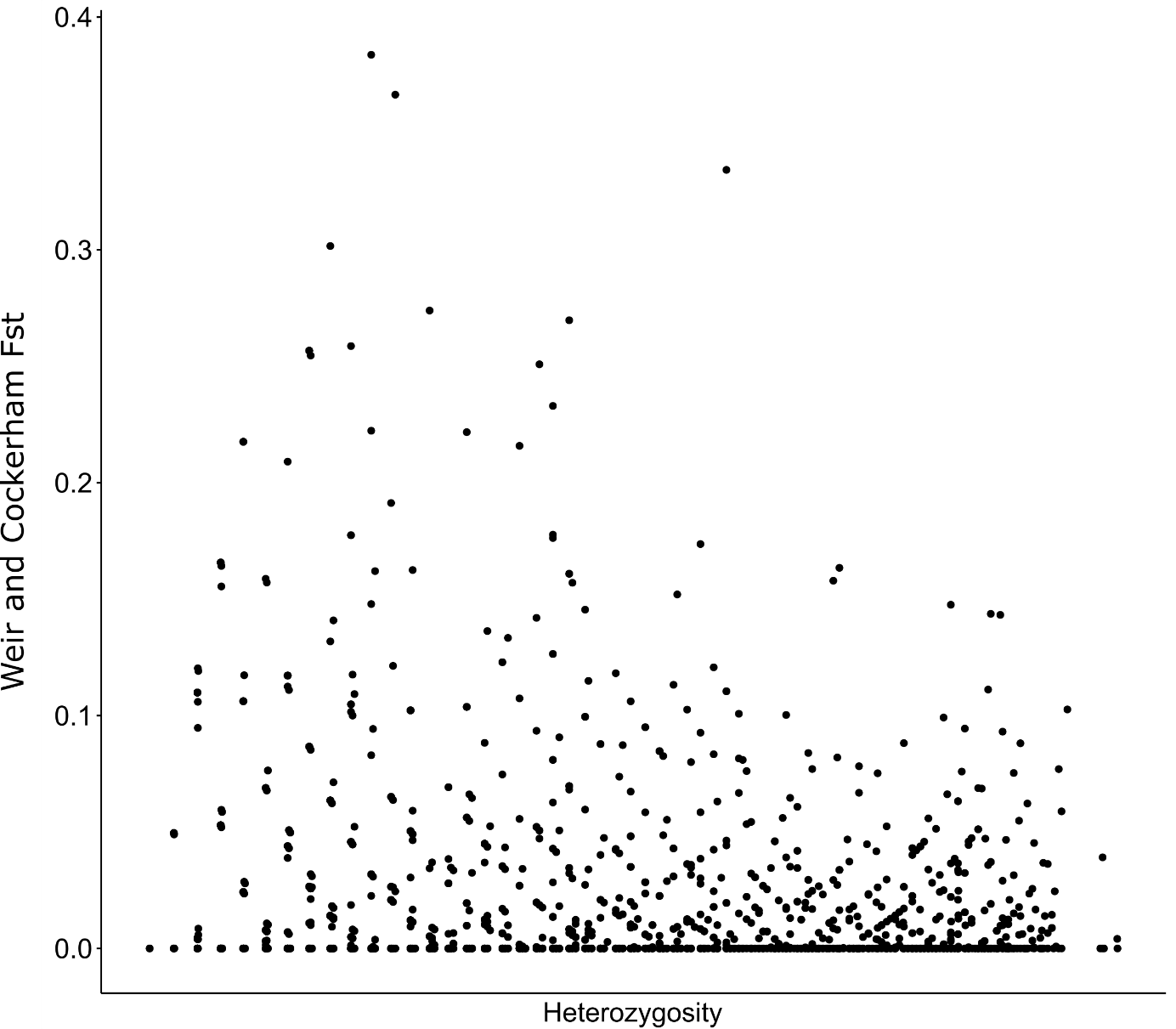

Supplement: Supplementary file 1 — Supplementary Information. [file 41598_2022_10529_MOESM1_ESM.docx]
